# Movable Kidney: Its Pathology, Symptoms, and Treatment

**Published:** 1909-12

**Authors:** 


**Movable Kidney: its Pathology, Symptoms, and Treatment.** By Harold W. Wilson, M.B., B.S. Lond., and C. M. Hinds Howell, M.B., B.Ch. Oxon. Pp. 104. London: Edward Arnold. 1908.

This work is based upon a careful study of the literature of its subject, upon original anatomical and experimental researches, and upon the clinical observation of cases treated in St. Bartholomew's Hospital in recent years.

In the introductory portion good definitions and a sound system of classification are suggested. Certain anatomical points bearing upon mobility of the kidney are then clearly and suggestively described. The authors incline to the view that the congenital mesonephron of floating kidney is "a myth of the embryologist's imagination," and that a mesentery, if present, is acquired as a result of the mobility of the organ.

The symptoms of acute and chronic cases of movable kidney are separately considered. We are surprised to learn that in a series of forty-one cases of nephrorrhaphy at St. Bartholomew's Hospital, no less than twenty-four were cases with acute symptoms, *i.e.* were characterised by intense pain and shock referable to mechanical obstruction either of the renal vessels or of the ureter, or of both these structures.

The authors, guided apparently by experiments upon cats, are strongly of opinion that nephrorrhaphy with partial decapsulation is much more likely to be successful than fixation without such decapsulation. We should attach more weight to this expression of opinion if clinical evidence had been adduced in its support.

Considered as a whole, the book contains much judicious criticism, an excellent description of the anatomy, pathology and symptomatology of movable kidney, and many records of cases which well illustrate the special points for which they have been selected from the literature of the subject, or from records hitherto unpublished.

